# CircITCH: A Circular RNA With Eminent Roles in the Carcinogenesis

**DOI:** 10.3389/fonc.2021.774979

**Published:** 2021-10-15

**Authors:** Soudeh Ghafouri-Fard, Tayyebeh Khoshbakht, Mohammad Taheri, Elena Jamali

**Affiliations:** ^1^ Department of Medical Genetics, School of Medicine, Shahid Beheshti University of Medical Sciences, Tehran, Iran; ^2^ Men’s Health and Reproductive Health Research Center, Shahid Beheshti University of Medical Sciences, Tehran, Iran; ^3^ Skull Base Research Center, Shahid Beheshti University of Medical Sciences, Tehran, Iran; ^4^ Department of Pathology, Loghman Hakim Hospital, Shahid Beheshti University of Medical Sciences, Tehran, Iran

**Keywords:** circular RNA, circITCH, cancer, expression, ncRNAs

## Abstract

Circular RNAs (circRNAs) are a group of long non-coding RNAs with enclosed structure generated by back-splicing events. Numerous members of these transcripts have been shown to affect carcinogenesis. Circular RNA itchy E3 ubiquitin protein ligase (circITCH) is a circRNA created from back splicing events in *ITCH* gene, a protein coding gene on 20q11.22 region. ITCH has a role as a catalyzer for ubiquitination through both proteolytic and non-proteolytic routes. CircITCH is involved in the pathetiology of cancers through regulation of the linear isoform as well as serving as sponge for several microRNAs, namely miR-17, miR-224, miR-214, miR-93-5p, miR-22, miR-7, miR-106a, miR-10a, miR-145, miR-421, miR-224-5p, miR-197 and miR-199a-5p. CircITCH is also involved in the modulation of Wnt/β-catenin and PTEN/PI3K/AKT pathways. Except from a single study in osteosarcoma, circITCH has been found to exert tumor suppressor role in diverse cancers. In the present manuscript, we provided a comprehensive review of investigations that reported function of circITCH in the carcinogenesis.

## Introduction

Circular RNAs (circRNAs) are a group of long non-coding RNAs with enclosed structure. This structure is created through establishment of a covalent bond between 5’ and 3’ termini through a back-splicing event in exons of a certain pre-mRNA (1). Several studies have indicated broad expression of circRNAs in mammalian cells in a cell type- or tissue-specific manner (1). CircRNAs have been shown to affect different cellular and biological processes, namely cell proliferation (1), differentiation, pluripotency (2) and epithelial-mesenchymal transition (EMT) (3). Moreover, they can participate in the remodeling of endoplasmic reticulum stress, autophagy and phagocytosis, DNA repair mechanisms as well as drug efflux (4). Different mechanisms have been suggested for circRNAs effects in these processes with the most appreciated one being their function as decoys for microRNAs (miRNAs) or RNA-binding proteins. Through this mechanism, circRNAs can influence expression of genes or translation of proteins with regulatory functions (1). CircRNA have the ability to base pair with other types of RNAs as well (5). Moreover, circRNAs can suppress activity of certain proteins, particularly cell cycle proteins through interacting with them (6). While circRNAs are mainly considered as non-coding RNAs, they might be served as a template for production of proteins under some conditions (5). Cumulatively, circRNAs can influence expression of cellular proteins, interfere with RNA-binding proteins to affect transcription of genes, regulate gene transcription *in cis*, and modulate splicing events (5). Yet, the competing endogenous function of circRNAs is the chief way through which they exert their biological effects (5). Several studies have emphasized on the role of circRNAs in cancer development and induction of chemo/radioresistance (4, 5).

Circular RNA itchy E3 ubiquitin protein ligase (circITCH) is an example of cancer-related circRNAs which can be used as target for therapeutic interventions. It is created from back splicing events in *ITCH* gene, a protein coding gene on 20q11.22 region. ITCH has a role as a catalyzer for ubiquitination through both proteolytic and non-proteolytic routes (7). It has been shown to affect tumorigenesis in a context-dependent manner (7). Recent studies have shown involvement of the circRNA from this locus in the carcinogenesis process. In the present manuscript, we provided a comprehensive review of investigations that reported function of circITCH in this process. The evidence regarding the role of circITCH in cancers is classified based on the samples/models used in the original papers to *in vitro*, *in vivo* and clinical studies.

## Cell Line Studies

### Bladder Cancer

CircITCH has been found to be down-regulated in bladder cancer cell lines. Forced over-expression of circITCH could inhibit proliferation, migratory potential, invasive properties and metastatic ability of bladder cancer cells. Functionally, circITCH acts as a sponge for miR-17 and miR-224 to up-regulate expression of their target genes p21 and PTEN. Cumulatively, circITCH functions as a tumor suppressor circRNA in bladder cancer (8).

### Breast Cancer

Expression of circITCH has also been shown to be decreased in triple negative breast cancer cell lines. Stable transfection of MDA-MB-231 and BT-549 cells with circITCH-expressing vectors has resulted in inhibition of proliferation, invasiveness and metastatic ability of these cells. Mechanistically, circITCH serves as a molecular sponge for miR-214 and miR-17 leading to enhancement of expression of the linear form of ITCH. This circRNA functionally inactivates Wnt/β-catenin signaling (9).

### Cervical Cancer

Expression of circITCH has also been shown to be down-regulated in cervical cancer cell lines. Up-regulation of circITCH in cervical cancer cells has inhibited their proliferation, migration, and invasiveness. Mechanistically, circITCH acts a sponge for miR-93-5p to regulate expression of FOXK2 (10).

### Osteosarcoma

Down-regulation of circITCH has also been verified in osteosarcoma cells. Overexpression of circITCH has induced cell apoptosis and decreased cell viability, proliferation, migratory potential and invasive properties of MG63 and Saos-2 osteosarcoma cells. This circRNA could decrease expression of miR-22 in osteosarcoma cells, thus suppressing PTEN/PI3K/AKT and SP-1 signals (11). On the other hand, another study in the hFOB1.19 osteoblast cell line and multiple osteosarcoma cell lines has shown up-regulation of circITCH in neoplastic cells compared with the osteoblast cells. Functionally, circITCH enhanced migration, invasive properties, and growth of these neoplastic cells through sponging miR-7 and increasing expression of EGFR (12).

**Figure 1:**
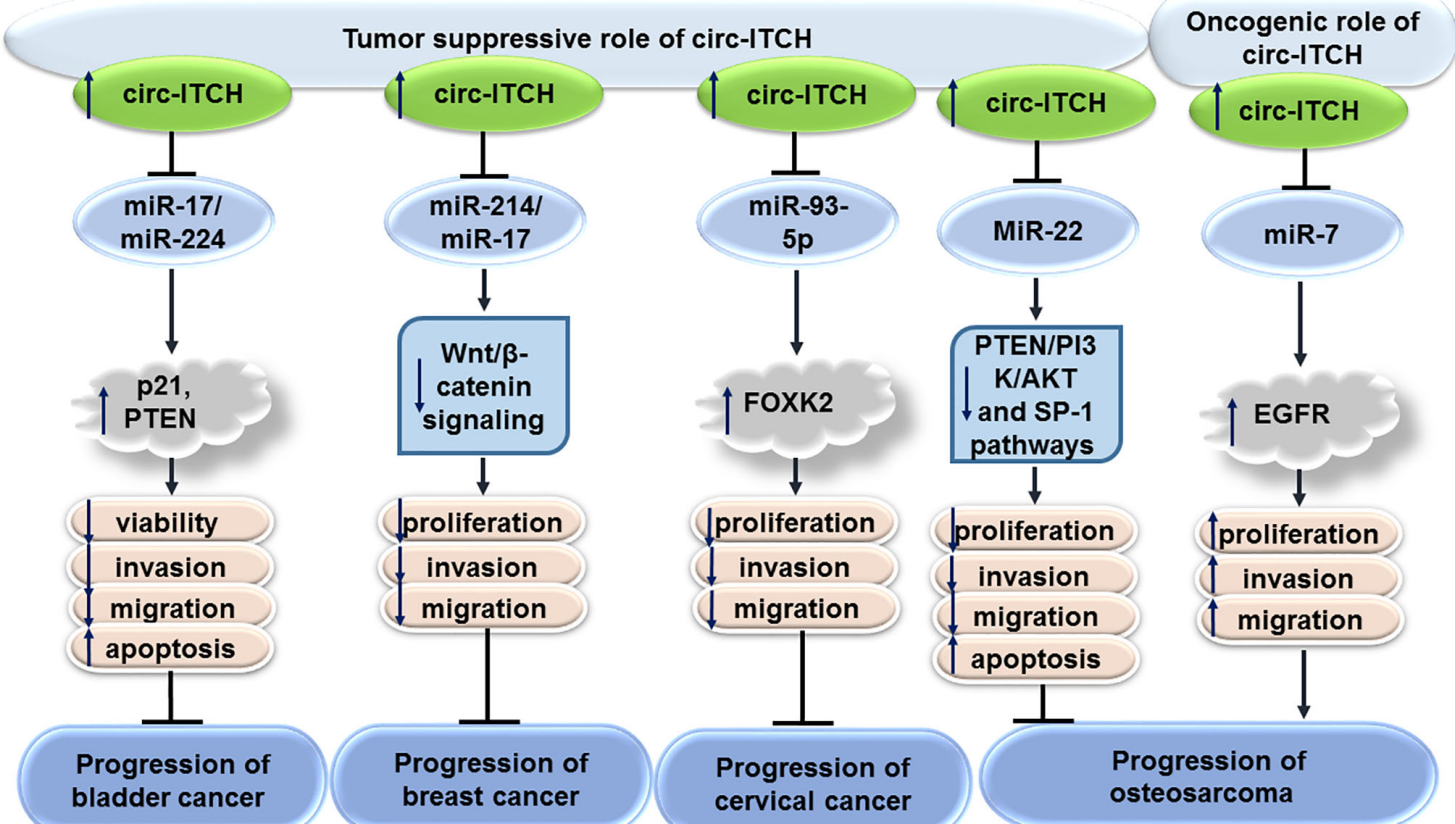
Tumor suppressor role of circITCH in bladder, breast and cervical cancers as well as dual role of this circRNA in osteosarcoma.

**Figure 1** shows the tumor suppressor role of circITCH in bladder, breast and cervical cancers as well as dual role of this circRNA in osteosarcoma.

### Thyroid Cancer

In thyroid cancer cells, forced over-expression of circITCH inhibits cell proliferation and invasive properties, while promoting cell apoptosis. These effects are mediated through sponging miR-22-3p and subsequent up-regulation of levels of CBL, an E3 ligase of nuclear β-catenin. Cumulatively, circITCH affects activity of the Wnt/β-catenin pathway through modulation of CBL levels, therefore suppressing progression of thyroid cancer (13).

### Ovarian Cancer

**Figure 2:**
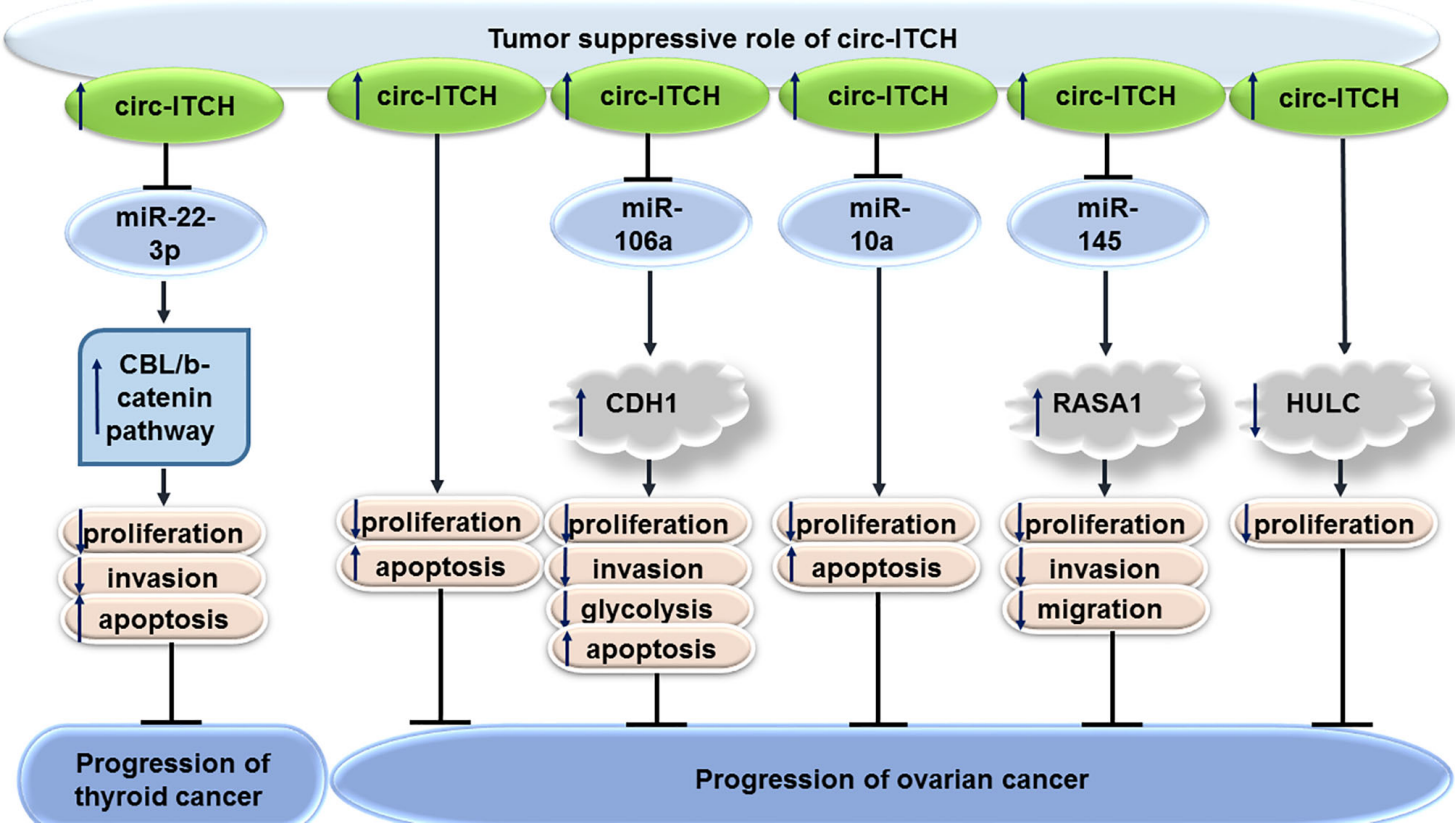
Tumor suppressor role of circITCH in thyroid and ovarian cancers.

Expression of circITCH expression has been found to be down-regulated in numerous epithelial ovarian cancer cell lines versus normal ovarian epithelial cells. This circRNA could inhibit proliferation of SKOV3 and OVCAR-3 cells, while enhancing their apoptosis (14). Another study has shown the role of circITCH in suppression of proliferation, invasiveness, and glycolytic process in ovarian cancer cells through sequestering miR-106a and enhancing expression of CDH1 (15). miR-10a-alpha has also been identified as a target of circITCH in ovarian cancer cells through which circITCH exerts its tumor suppressor effects (16). Moreover, circITCH has been shown to suppress progression of this cancer *via* influencing miR-145/RASA1 axis (17). Finally, circITCH has been suggested to suppress proliferation of ovarian cells through deceasing expression of HULC (18). **Figure 2** shows the tumor suppressor role of circITCH in thyroid and ovarian cancers.

### Hepatocellular Carcinoma

CircITCH has also been shown to have tumor suppressor roles in hepatocellular carcinoma. In fact, the effects of lidocaine on inhibition of proliferation of hepatocellular carcinoma cells have been shown to be mediated through restoration of circITCH in these cells. Mechanistically, circITCH modulates expression of CPEB3 through sponging miR-421 (19). CircITCH can also affect progression of hepatocellular carcinoma through sponging miR-224-5p and increasing expression of MafF (20). CircRNAITCH levels have been found to down-regulated in several hepatocellular cancer cell lines compared with normal hepatic L-02 cell line. Up-regulation of circRNAITCH has inhibited proliferation of these cells, suppressed their colony formation aptitude and induced their apoptosis. CircRNAITCH could be used as a sponge for miR-7 and miR-214. Through this route, it regulates Wnt/β-catenin signals and suppresses c-myc and cyclin D1 levels (21).

### Glioma

CircITCH has also been shown to inhibit proliferation and invasive potential of glioma cells *via* sequestering miR-106a-5p and enhancing expression of SASH1 (22). Moreover, it has been reported to serve as a sponge for miR-214 and promote expression of linear ITCH in glioma cells (23).

### Oral Squamous Cell Carcinoma

The miR-421/PDCD4 axis has been shown as the downstream axis mediating the role of circITCH in modulation of progression of oral squamous cell carcinoma by regulating (24).

**Figure 3:**
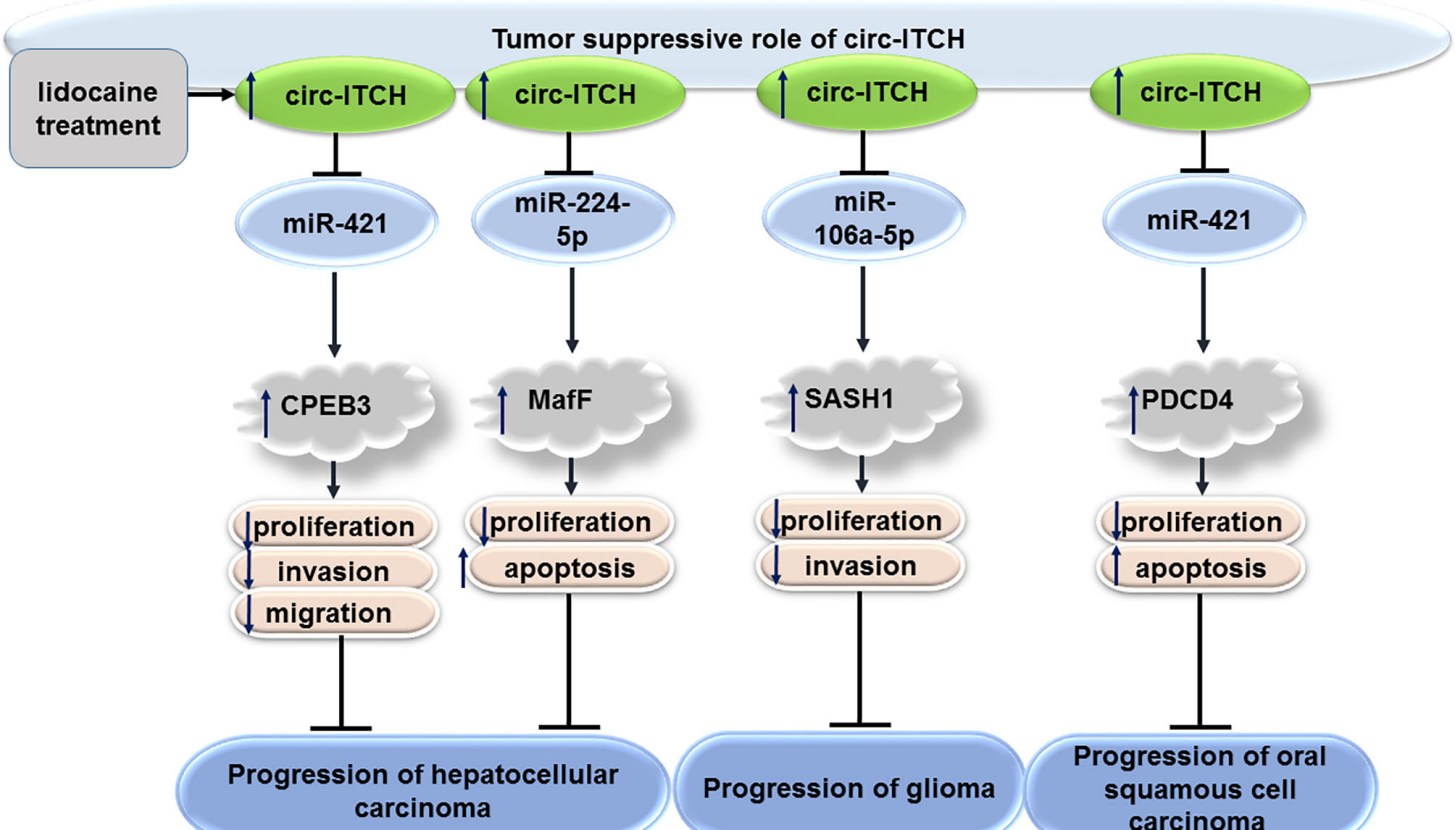
Tumor suppressor role of circITCH in hepatocellular carcinoma, glioma and oral squamous cell carcinoma.

**Figure 3** shows the tumor suppressor role of circITCH in hepatocellular carcinoma, glioma and oral squamous cell carcinoma.

### Prostate Cancer

CircITCH exerts tumor suppressor roles in prostate cancer *via* influencing miR-17-5p/HOXB13 axis (25). Moreover, circITCH can inhibit proliferation, migratory aptitude, and invasiveness of human prostate cancer cells through sequestering miR-17. This circRNA can also down-regulate expression levels of several proteins in the Wnt/β-catenin and PI3K/AKT/mTOR signal transductions in LNCaP and PC-3 cells, as representatives of androgen receptor (AR)-positive and AR-negative cells, respectively (26). miR-197 is another target of circITCH in prostate cancer cells through which it regulates progression of this type of cancer (27).

### Gastric Cancer

In addition, circITCH can suppress gastric carcinogenesis through modulation of miR-199a-5p/Klotho axis (28) as well as the Wnt/β-catenin pathway (29).

### Melanoma

Finally, circITCH decreases expression of GLUT1 and inhibits uptake of glucose by melanoma cells to suppress their proliferation (30).

**Figure 4:**
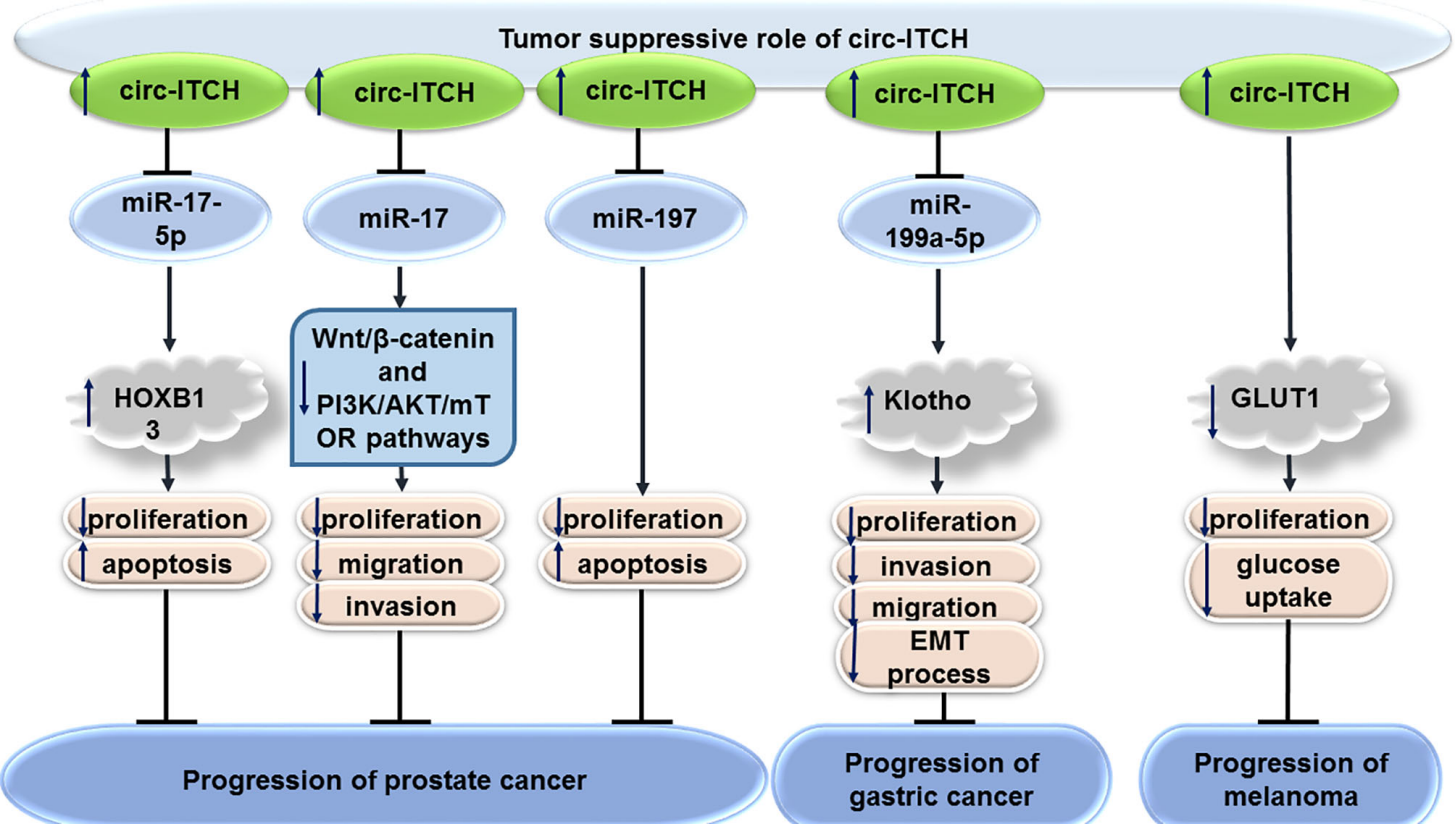
Tumor suppressor role of circITCH in prostate cancer, gastric cancer and melanoma.

**Figure 4** shows the tumor suppressor role of circITCH in prostate cancer, gastric cancer and melanoma.

### Other Cancers

miR-7 and miR-214 have been found to be sequestered by circITCH in lung (31) and esophageal cancers (32). In addition to mentioned cancer types, circITCH has tumor suppressor roles in renal cancer (33), multiple myeloma (34) and colorectal cancer (35). **Table 1** summarizes expression and function of circITCH in cancer cell lines

**Table 1:** Expression and function of circITCH in cancer cell lines (∆: knock-down or deletion, BTZ: Bortezomib).

Tumor type	Targets/Regulators and Signaling Pathways	Cell line	Function	Reference
Bladder cancer	miR-17, miR-224, p21, PTEN	EJ, T24	↑ circITCH: ↓ viability, ↓ migration, ↓ invasion, ↑ G1/S cell cycle arrest, ↑ apoptosis	(8)
Breast cancer	miR-214, miR-17, Wnt/β-catenin signaling	MCF-10A, MCF-7, T47D, SK-BR-3, MDA-MB-231, BT-549	↑ circITCH: ↓ proliferation, ↓ migration, ↓ invasion	(9)
Cervical cancer	miR-93-5p, FOXK2	HeLa	↑ circITCH: ↓ proliferation, ↓ migration, ↓ invasion	(10)
Osteosarcoma	miR-22, PTEN/PI3K/AKT and SP-1 pathways	MG63, U2OS, Saos-2, hFOB1.19	↑ circITCH: ↓ proliferation, ↓ migration, ↓ invasion, ↑ apoptosis	(11)
	miR-7, EGFR	SJSA-1, U2OS, hFOB1.19	↑ circITCH: ↑ proliferation, ↑ migration, ↑ invasion	(12)
Thyroid cancer	miR-22-3p, CBL/b-catenin pathway	K1, IHH4, TPC1	↑ circITCH: ↓ proliferation, ↓ invasion, ↑ apoptosis	(13)
Ovarian cancer	_	SKOV3, OVCAR-3	↑ circITCH: ↓ proliferation, ↑ apoptosis	(14)
	miR-106a, CDH1	A2780 and OVCAR3, ISOE80	↑ circITCH: ↓ proliferation, ↓ invasion, ↓ glycolysis, ↑ apoptosis	(15)
	miR-10a	SKOV3, A-2780, OVCAR-3, HO-8910, IOSE80	↑ circITCH: ↓ proliferation, ↑ apoptosis	(16)
	miR-145, RASA1	SK-OV-3, Caov-3	↑ circITCH: ↓ proliferation, ↓ migration, ↓ invasion	(17)
	HULC	UWB1.289 + BRCA1 and UWB1.289	↑ circITCH: ↓ proliferation	(18)
Hepatocellular carcinoma	miR-421, CPEB3	Huh7, Hep3B, THLE-2	∆ circITCH: ↓ suppressive effect of lidocaine on hepatocellular carcinoma development lidocaine treatment: ↑ circ-ITCH, ↓ proliferation, ↓ migration, ↓ invasion	(19)
	miR-224-5p, MafF	SMMC7721, Huh7, Hep3B	↑ circITCH: ↓ proliferation, ↑ apoptosis, ↑ MafF levels	(20)
	miR-7, miR-214, Wnt/βcatenin signaling	HCC Huh-7, U251, HB611, SMMC-7721, L-02	↑ circITCH: ↓ proliferation, ↓colony formation ability, ↑ apoptosis,	(21)
Glioma	miR-106a-5p, SASH1	U251, U87, SHG44, A172, HEB	↑ circITCH: ↓ proliferation, ↓ invasion	(22)
	miR-214, linear ITCH, Wnt/β-catenin pathway	U87, U251, A172, SHG44, LN229, T98G, SHG139, M059K	↑ circITCH: ↓ proliferation, ↓ migration, ↓ invasion, ↓ EMT process, ↑ apoptosis	(23)
Oral squamous cell carcinoma	miR-421, PDCD4	HOK, SCC6, SCC9, SCC25, HN4, HN6	↑ circITCH: ↓ proliferation, ↑ apoptosis	(24)
Prostate cancer	miR-17-5p, HOXB13	C4-2, LNCaP, DU145, 22Rv1, PC-3 and VcaP, RWPE-1	↑ circITCH: ↓ proliferation, ↑ apoptosis	(25)
	miR-17, Wnt/β-catenin and PI3K/AKT/mTOR pathways	RWPE-1, LNCaP, PC-3	↑ circITCH: ↓ proliferation, ↓ migration, ↓ invasion	(26)
	miR-197	U 145, 22RV1, VCaP, PC-3, RWPE	↑ circITCH: ↓ proliferation, ↑ apoptosis	(27)
Gastric cancer	miR-199a-5p, Klotho	HGC-27, AGS, MKN-45, MGC-803 and HEK-293 T, GES-1	↑ circITCH: ↓ proliferation, ↓ migration, ↓ invasion, ↓ EMT process, effect on anticancer chemotherapy	(28)
	miR-17, ITCH, Wnt/β-catenin pathway	GES-1, AGS, MKN45	↑ circITCH: ↓ proliferation, ↓ migration, ↓ invasion	(29)
Melanoma	GLUT1	A375, M21	↑ circITCH: ↓ proliferation, ↓ glucose uptake	(30)
	miR-520f	A375, WM35	∆ circITCH: ↑ proliferation, ↑colony-forming ability	(36)
Lung cancer	miR-7 and miR-214, linear ITCH, Wnt/β-catenin signaling	A549, NIC-H460	↑ circITCH: ↓ proliferation	(31)
Esophageal squamous cell carcinoma	miR-7, miR-17, miR-214, ITCH, Wnt/β-catenin pathway	Eca-109, TE-1	↑ circITCH: ↓ proliferation, ↓colony-forming ability	(32)
Clear cell renal cell carcinoma	miR-106b-5p, PDCD4	HK-2, OSRC-2, A498, SW839, 786-O, Caki-1, GRC-1	↑ circITCH: ↓ metastasis, ↓ migration, ↓ invasion	(33)
Multiple myeloma	miR-615-3p, PRKCD	U-266, NCI-H929, RPMI 8226, NCI-H929, RPMI 8226	↑ circITCH: ↓ proliferation, ↑ apoptosis, ↑ sensitivity to BTZ	(34)
Colorectal cancer	Linear ITCH, Wnt/β-catenin pathway	HCT116, SW480	↑ circITCH: ↓ proliferation	(35)

## Animal Studies

Subcutaneous injection of T24 bladder cancer cells transfected with circITCH into the nude mice has shown the impact of this circRNA in reduction of tumor volumes and tumor weight. Notably, expressions of p21 and PTEN have been up-regulated in the tumors originated from circITCH overexpressing cells (8). Other *in vivo* studies have consistently verified the tumor suppressor roles of circITCH in different animal models (**Table 2**). Similarly, over-expression of circITCH has increased sensitivity of bortezomib-resistant multiple myeloma cells to this drug in animal models (34).

**Table 2:** Summary of studies which assessed impact of circITCH up-regulation or silencing in animal models (∆: knock-down or deletion, BTZ: Bortezomib).

Tumor Type	Animal models	Results	Reference
Bladder cancer	female athymic BALB/C nude mice	↑ circITCH: ↓ tumor volume, ↓ tumor weight	(8)
Breast cancer	female BALB/c nude mice	↑ circITCH: ↓ tumor volume, ↓ number of lung nodules	(9)
Cervical cancer	nude mice	↑ circITCH: ↓ tumorigenesis	(10)
Thyroid cancer	female BALB/c nude mice	↑ circITCH: ↓ tumor size, ↓ tumor weight	(13)
Ovarian cancer	BALB/c nude mice	↑ circITCH: ↓ tumor volume, ↓ tumor weight	(15)
	female BALB/c nude mice	↑ circITCH: ↓ tumor volume, ↓ tumor weight	(17)
Hepatocellular carcinoma	male BALB/c nude mice	∆ circITCH: ↑ tumor volume, ↑ tumor weight under lidocaine treatment condition	(19)
Glioma	BALB/c nude mice	↑ circITCH: ↓ tumor growth, ↓ tumor weight	(22)
Prostate cancer	female BALB/c nude mice	↑ circITCH: ↓ tumor growth, ↓ tumor volume	(25)
Gastric cancer	male athymic nude mice	↑ circITCH: ↓ tumor growth	(29)
Esophageal squamous cell carcinoma	female BALB/c nude mice	↑ circITCH: ↓ tumor growth	(32)
Clear cell renal cell carcinoma	male BALB/c mice	↑ circITCH: ↓ tumor volume, ↓ tumor weight	(33)
Multiple myeloma	BALB/c nude mice	↑ circITCH + BTZ treatment: ↓ tumor volume	(34)

## Clinical Studies

Different studies in samples obtained from patients with diverse types of neoplasms have verified down-regulation of circITCH in neoplastic samples when compared with normal (non-affected) tissues (**Table 3**). Down-regulation of circITCH in bladder cancer tissues has been correlated with histological grade. In addition, bladder cancer patients who had circITCH down-regulation exhibited poor clinical outcome (8).

**Table 3:** Results of studies that reported dysregulation of circITCH in clinical samples (ANCTs, adjacent non-cancerous tissues; OS, Overall survival; FIGO, International Federation of Gynecology and Obstetrics; DFS, disease-free survival; TNM, tumor‐node‐metastasis).

Tumor type	samples	Expression (Tumor vs. Normal)	Kaplan-Meier analysis (impact of circITCH down-regulation)	Univariate/Multivariate cox regression	Association of down-regulation of circITCH with clinicopathologic characteristics	Reference
Bladder cancer	72 pairs of tumor tissues and ANCTs	down	shorter OS	_	histological grade	(8)
Breast cancer	275 tumor tissues and 68 ANCTs	down	shorter OS	_	lymph node metastasis, larger tumor size and advanced TNM stage	(9)
Osteosarcoma	22 pairs of osteosarcoma tissues and para- osteosarcoma tissues	down	_	_	_	(11)
Thyroid cancer	37 tumor tissues and 14 ANCTs	down	_	_	clinical stage and lymph node metastasis	(13)
Ovarian cancer	77 tumor tissues and ANCTs	down	shorter OS	CircITCH was found to be an independent predictive factor for favorable OS.	Larger tumor size, increased FIGO stage	(14)
	45 pairs of tumor tissues and ANCTs	down	shorter 5-year OS	_	Larger tumor size, increased FIGO stage	(15)
	20 pairs of tumor tissues and ANCTs	down	shorter OS	_	_	(17)
	75 pairs of tumor tissues and ANCTs	down	_	_	_	(18)
Hepatocellular carcinoma	40 tumor tissues and 34 ANCTs	down	_	_	_	(19)
	288 pairs of tumor tissues and ANCTs	down	shorter OS	_	_	(37)
Glioma	48 pairs of tumor tissues and ANCTs	down	_	_	_	(22)
	60 pairs of tumor tissues and ANCTs	down	poor OS	_	tumor size, WHO grade and Karnofsky Performance Status	(23)
Oral squamous cell carcinoma	103 pairs of tumor tissues and ANCTs	down	shorter OS	_	lymph node metastasis and advanced TNM stage	(24)
Prostate cancer	52 pairs of tumor tissues and ANCTs	down	poor OS	_	preoperative PSA, Gleason score, and tumor stage	(25)
	10 pairs of tumor tissues and ANCTs	down	_	_	_	(26)
	324 pairs of tumor tissues and ANCTs	down	shorter DFS and OS	_	advanced pathologic T stage and high risk of lymph node metastasis	(38)
Gastric cancer	61 pairs of tumor tissues and ANCTs	down	_	_	invasion depth	(28)
	30 pairs of tumor tissues and ANCTs	down	_	_	age and tumor grades	(39)
	51 pairs of tumor tissues and ANCTs	down	poor OS	_	lymph node metastasis	(29)
Melanoma	56 pairs of tumor tissues and ANCTs	down	_	_	_	(30)
Lung cancer	78 pairs of tumor tissues and ANCTs	down	_	_	age	(31)
Esophageal squamous cell carcinoma	684 pairs of tumor tissues and ANCTs	down	_	_	_	(32)
Clear cell renal cell carcinoma	54 pairs of tumor tissues and ANCTs	down	_	_	_	(33)
Multiple myeloma	56 patients with MM and 56 HCs	down	shorter OS	_	_	(34)

Expression of circITCH has also been reported to be lower in ovarian tumor tissues compared with corresponding non-tumoral tissues. Most notably, expression of circITCH has been inversely correlated with tumor size and FIGO stage in these patients. Based on multivariate Cox analyses, over-expression of circITCH has been identified as an independent predictor of favorable overall survival of patients with ovarian cancer (14).

Cumulatively, decreased levels of circITCH have been correlated with poor outcome in diverse types of cancers, suggesting this circRNA as a prognostic factor in human malignancies.

## Discussion

Except from a single study in osteosarcoma, circITCH has been found to exert tumor suppressor role in diverse cancers. This circRNA is involved in the pathetiology of cancers through regulation of the linear isoform as well as serving as sponge for several microRNAs, namely miR-17, miR-224, miR-214, miR-93-5p, miR-22, miR-7, miR-106a, miR-10a, miR-145, miR-421, miR-224-5p, miR-197 and miR-199a-5p. CircITCH also partakes in the modulation of Wnt/β-catenin and PTEN/PI3K/AKT pathways.

A number of miRNAs have been found to interact with circITCH in diverse tissues. For instance, miR-7 has been found to be sponged by circITCH in osteosarcoma, hepatocellular carcinoma, lung cancer and esophageal squamous cell carcinoma. Meanwhile, miR-17 has been detected as a target of this circRNA in bladder, breast, prostate, gastric and esophageal squamous cell cancers. Moreover, circITCH has been shown to sponge miR-214 in breast, lung, hepatocellular carcinoma, glioma and esophageal cancers. Thus, circITCH/miR-7, circITCH/miR-17 and circITCH/miR-214 axes are appropriate therapeutic targets for diverse types of cancers.

The correlation between expression levels of circITCH and clinicopathological data such as tumor size, local invasion, distant metastasis and different staging systems shows the importance of this circRNA in the development or progression of cancers, representing a novel biomarker role for it. Although the impact of circITCH in determination of prognosis of cancer patients is well established, its function as a diagnostic marker is not studied. Since circRNAs are stable transcripts in the circulation, they are expected to reflect cancer course. Thus, future investigations should focus on evaluation of levels of circITCH in plasma of patients with different stages of cancers to find the suitability of this marker for diagnostic purposes as well as patients’ follow-up. The main question in this regard is whether expression level of circITCH is changed after chemo/radiotherapy or tumor excision. If so, it can be used as a marker for early detection of cancer recurrence.

Another question to be answered is the correlation between expression levels of the circular and linear form of ITCH in different types of cancers. The answer to this question can help in better understanding of the mechanism of dysregulation of circITCH in relation to cancer progression.

Since circITCH is mostly considered as a tumor suppressor circRNA, several groups have assessed the impact of forced over-expression of this transcript in cancer cells transplanted into animal models. The results have been mostly promising, yet needing to be approved in clinical settings.

The interactions between circITCH and RNA-binding proteins have not identified in the previous literature. However, the online database circular RNA Interactome (https://circinteractome.nia.nih.gov/) has listed a number of RNA-binding proteins possibly interacting with circRNAs originated from *ITCH* locus (**Table 4**).

**Table 4:** Possible interactions between circITCH and RNA-binding proteins.

CircRNA ID	RNA-binding protein sites matching circRNA junction	RNA-binding protein sites matching flanking regions of circRNA
hsa_circ_0001141	–	EIF4A3, HuR, U2AF65
hsa_circ_0003073	–	DGCR8, EIF4A3, PTB
hsa_circ_0005677	EIF4A3	EIF4A3, PTB
hsa_circ_0005868	–	AGO2, EIF4A3, PTB, U2AF65

## Future Perspectives

CircITCH, as a tumor suppressor circRNA can be utilized in therapeutic regimens for cancers. Delivery methods include nanoparticles and exosome-based methods (40). Artificial circRNAs have been successfully used as miRNA sponges in recent years (41). Thus, synthetic circITCH molecules with the potential of sponging oncogenic miRNAs can be used for attenuation of carcinogenic process. Yet, this method should be appraised in cell lines and animal models. Finally, the interactions between circITCH and RNA-binding proteins should be assessed in future investigations.

## Author Contributions

SG-F wrote the draft and revised it. MT designed and supervised the study. TK and EJ collected the data and designed the tables and figures. All authors contributed to the article and approved the submitted version.

## Conflict of Interest

The authors declare that the research was conducted in the absence of any commercial or financial relationships that could be construed as a potential conflict of interest.

## Publisher’s Note

All claims expressed in this article are solely those of the authors and do not necessarily represent those of their affiliated organizations, or those of the publisher, the editors and the reviewers. Any product that may be evaluated in this article, or claim that may be made by its manufacturer, is not guaranteed or endorsed by the publisher.
